# mRNA as a Tool for Gene Transfection in 3D Cell Culture for Future Regenerative Therapy

**DOI:** 10.3390/mi11040426

**Published:** 2020-04-18

**Authors:** Satoshi Uchida, Kayoko Yanagihara, Akitsugu Matsui, Kazunori Kataoka, Keiji Itaka

**Affiliations:** 1 Department of Bioengineering, Graduate School of Engineering, The University of Tokyo, Bunkyo, Tokyo 113-8656, Japan; 2 Innovation Center of NanoMedicine (iCONM), Kawasaki Institute of Industrial Promotion, Kawasaki, Kanagawa 210-0821, Japan; kayofuru10@yahoo.co.jp (K.Y.); m_akirin@yaoo.co.jp (A.M.); k-kataoka@kawasaki-net.ne.jp (K.K.); 3 Institute for Future Initiatives, The University of Tokyo, Bunkyo, Tokyo 113-0033, Japan; 4 Department of Biofunction Research, Institute of Biomaterials and Bioengineering, Tokyo Medical and Dental University (TMDU), Chiyoda, Tokyo 101-0062, Japan

**Keywords:** mRNA therapeutics, 3D cell culture, mesenchymal stem cell, regenerative therapy, osteogenesis, polycation

## Abstract

A combination of three-dimensional (3D) cell culturing and non-viral gene transfection is promising in improving outcomes of cell transplantation therapy. Herein, gene transfection profiles in 3D cell culture were compared between plasmid DNA (pDNA) and messenger RNA (mRNA) introduction, using mesenchymal stem cell (MSC) 3D spheroids. Green fluorescence protein (GFP) mRNA induced GFP protein expression in 77% of the cells in the spheroids, whereas only 34% of the cells became GFP positive following pDNA introduction. In mechanistic analyses, most of the cells in MSC spheroids were non-dividing, and pDNA failed to induce GFP expression in most of the non-dividing cells. In contrast, both dividing and non-dividing cells became GFP-positive after mRNA introduction, which led to a high overall percentage of GFP-positive cells in the spheroids. Consequently, mRNA encoding an osteogenic factor, runt-related transcription factor 2 (Runx2), allowed in vitro osteogenic differentiation of MSCs in spheroids more efficiently compared to Runx2 pDNA. Conclusively, mRNA exhibits high potential in gene transfection in 3D cell culture, in which the cell division rate is lower than that in monolayer culture, and the combination of mRNA introduction and 3D cell culture is a promising approach to improve outcomes of cell transplantation in future regenerative therapy.

## 1. Introduction

Cell transplantation therapy has demonstrated excellent potential in a variety of medical fields, both in preclinical and clinical research [1,2,3]. Transplanted cells exert their therapeutic effect by integrating into host tissues and replacing endogenous cell functions [4], and additionally by secreting paracrine factors [5]. Strategies to improve these effects are vigorously being developed. For example, three-dimensional (3D) culturing is effective in preserving physiological function of cells during ex vivo culturing, and also in improving engraftment efficiency and therapeutic functionalities of cells in host tissues following transplantation [6,7]. Genetic modification of transplanted cells is also a promising approach, utilizing various types of factors including genome editing enzymes, chimeric antigen receptors, and factors for (de-)differentiation, paracrine signaling, pro-survival effect and cell adhesion [8,9,10]. Combinational use of 3D culturing and genetic modification has also been attempted, which has provided synergistic effects [11,12,13,14].

Regarding the introduction of genetic materials to cells for transplantation therapy, viral gene transduction has several potential issues, including complicated manufacturing processes, difficulty in scale-up, high economic costs, limitation in the size of genes, safety concerns, and immune attack to transplanted cells expressing viral proteins [15,16,17,18]. Non-viral transfection of plasmid DNA (pDNA) or messenger RNA (mRNA) is a promising option to address these issues. In transfection of 3D cultured cells, transfection capability to non- or slowly-dividing cells is critical, as the cell cycle in 3D culture tends to be slower compared to that in monolayer cultures [19,20]. In this regard, introduction of pDNA to non-dividing cells is inefficient, because nuclear entry of pDNA is inhibited by the nuclear membrane except when the membrane disappears during cell division [21,22]. In contrast, mRNA is capable of inducing efficient protein expression even in non-dividing cells [23,24], which motivates us to use mRNA for gene introduction in 3D cultured cells.

In this study, we investigated the protein expression profile in 3D cultured cells after introducing mRNA and pDNA, by focusing on the relationship between cell division and protein expression. For 3D culturing, micropatterned plates, comprising an array of 100 μm-sized cell adhesive areas surrounded by a non-cell adhesive area, were used, which allowed us to prepare a large number of spheroids with a uniform diameter of 100 μm [25,26]. For introduction of mRNA and pDNA, poly[*N*’-[*N*-(2-aminoethyl)-2-aminoethyl]aspartamide] [PAsp(DET)] polycation was employed, which has two notable features: pH-responsive protonation behavior allows for efficient endosomal escape of polyplexes and its biodegradable nature contributed to its low cumulative toxicity [27,28,29]. Using these platforms, we showed that mRNA introduction provided protein expression in a larger percentage of cells in mesenchymal stem cell (MSC) spheroids compared to introduction of pDNA, leading to enhanced outcomes after in vitro osteogenic induction using mRNA encoding an osteogenic transcription factor.

## 2. Materials and Methods 

### 2.1. Spheroid Preparation

Human MSCs (Lonza, Basel, Switzerland) were cultured using Dulbecco’s modified Eagle’s medium (DMEM) containing 10% fetal bovine serum (FBS, HyClone Laboratories, GE Healthcare Life Science, South Logan, UT, USA) and 1% penicillin/streptomycin (Sigma–Aldrich, St. Louis, MO, USA). MSCs at passage 5 were used for the experiments. For spheroid preparation, MSCs were seeded onto micropatterned plates (Cell-able^TM^, Toyo Gosei, Tokyo, Japan) at a density of 400,000 cells/well in 12-well plates and 40,000 cells/well in 96 well plates. A bright-field image of spheroids was obtained with an all-in-one fluorescence microscope BZ-X710 (Keyence, Osaka, Japan).

### 2.2. Preparation of mRNA and pDNA

Enhanced GFP (EGFP) mRNA was purchased from Trilink (San Diego, CA, USA). Runt-related transcription factor 2 (Runx2) mRNA was prepared by in vitro transcription (IVT) as described previously [30]. Briefly, a flag-tagged mouse Runx2 DNA sequence, a kind gift from K. Miyazono (The University of Tokyo, Tokyo, Japan), was inserted into a pSP73 vector (Promega, Madison, WI, USA) possessing a 120 bp poly A/T sequence. IVT was performed using a mMESSAGE mMACHINE T7 ULTRA Kit (Ambion, Invitrogen, Carlsbad, CA, USA), followed by purification using an RNeasy mini kit (QIAGEN, Hilden, Germany) and spectroscopic measurement of concentration at 260 nm. Purity of mRNA was assessed spectroscopically based on ultraviolet (UV) absorption, and size of mRNA was evaluated by on-chip capillary electrophoresis using Bioanalyzer Agilent2100 (Agilent, Santa Clara, CA, USA). For pDNA construction, a sequence coding EGFP (Clontech, Palo Alto, CA, USA) and that coding a flag-tagged mouse Runx2 were inserted into pCAG-GS vectors (RIKEN, Tokyo, Japan). Notably, mRNA and pDNA were designed to possess the same protein coding sequences in both cases of EGFP (Genbank: JA532579.1) and Runx2 (RefSeq: NM_001145920.2).

### 2.3. Transfection of MSCs with GFP mRNA and pDNA

PAsp(DET) was synthesized as previously described [27]. Using ^1^H-NMR, the polymerization degree of PAsp(DET) was determined to be 52. PAsp(DET) and mRNA or pDNA was mixed at a residual molar ratio of amine groups in polycations to phosphate groups in nucleic acids (N/P) of 10.

MSC spheroids were used for transfection 3 days after seeding of MSCs onto 96-well micropatterned culture plates. Culture medium was replaced with serum-free Opti-MEM medium (Gibco, Thermo Fisher Scientific, Waltham, MA, USA), and 0.3 μg of mRNA or pDNA, complexed with PAsp(DET), was added to each well. Six hours after the transfection, the medium was replaced with DMEM containing 10% FBS and 1% penicillin/streptomycin. Eighteen hours later, cell nuclei were stained using Hoechst 33342 (Dojindo Laboratories, Kumamoto, Japan), and confocal laser scanning microscopy (CLSM) imaging was subsequently performed using an LSM 510 instrument (Carl Zeiss, Oberlochen, Germany) with a 10× objective at excitation wavelengths of 488 nm for GFP and 710 nm (MaiTai laser for 2-photon imaging) for Hoechst 33342. Images were analyzed quantitatively using IN Cell developer tool box software (GE Healthcare, Buckinghamshire, UK). For 5-ethynyl-2’-deoxyuridine (EdU) staining of dividing cells, MSCs were cultured in the presence of EdU at a final concentration of 100 μM for 24 h after addition of mRNA or pDNA. Then, EdU was stained using a Click-iT EdU Alexa Fluor 647 imaging kit, with GFP immunostaining using GFP-booster (Chromotek GmbH, Planegg-Martinsried, Germany), according to manufacture’s protocol, followed by CLSM observation at an excitation wavelength of 633 nm for EdU, 488 nm for GFP, and 710 nm (2-photon laser) for Hoechst 33342. For quantification of the total GFP expression level, cells were lysed with passive lysis buffer (Promega, Madison, WI), followed by fluorescence measurement of the lysate using a microplate reader infinite M1000 PRO (Tecan Group Ltd., Männedorf, Switzerland).

For transfection to monolayer cultured cells, MSCs were seeded onto 96-well culture plates at the density of 5000 cells/well. After 24 h of culturing, transfection was performed as described above for MSC spheroids. Twenty-four hours after addition of GFP pDNA or mRNA, fluorescence images were obtained with an all-in-one fluorescence microscope BZ-X710, followed by quantitative image analyses as described above.

In all of these transfection experiments, weight rather than molar amount was controlled to be the same between mRNA and pDNA. Because the toxicity of transfection reagents is a major dose-limiting factor in non-viral gene transfection, it is practically reasonable to use comparable dose of transfection reagents in both mRNA and pDNA transfection. Further notably, charge ratio of polycations and nucleic acids is a determinant of polyplex transfection processes, including cellular uptake and endosomal escape of the polyplex, and thus should be controlled to be the same between mRNA and pDNA transfection. Thus, the same weight of mRNA and pDNA was used for transfection.

### 2.4. In Vitro Osteogenic Differentiation

For alkaline phosphatase (ALP) measurement, 0.3 μg of Runx2 mRNA or pDNA was transfected to MSCs cultured in 96-well plates, four times every 3 days. Fourteen days after the first transfection, ALP expression was measured using a TRACP & ALP assay kit (Takara Bio Inc., Shiga, Japan), which uses p-nitro-phenyl phosphate substrate to detect ALP enzymatic activity spectroscopically at an absorbance of 405 nm. For osteocalcin transcript measurement, 3 μg of Runx2 mRNA or pDNA was transfected to MSCs cultured in 12-well plates, four times every 3 days. Twenty-one days after the first transfection, total RNA in MSCs was extracted using an RNeasy mini kit, followed by quantitative real-time PCR using an ABI Prism 7500 sequence detector (Applied Biosystems, Foster City, CA, USA), and TaqMan gene expression assays (Applied Biosystems; Hs01587814_g1 for osteocalcin and Hs01060665_g1 for β-actin). In these osteogenic differentiation experiments, MSCs were cultured in MSC Go Rapid Osteogenic XF (Biological Industries, Beit Haemek, Israel), after the first transfection.

## 3. Results

**Figure 1:**
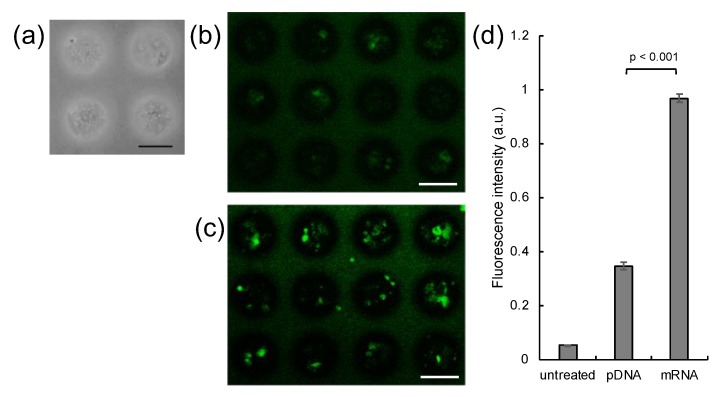
Introduction of GFP pDNA and mRNA to MSC spheroids. (**a**) MSC spheroids were prepared 3 days after seeding MSCs onto a micropatterned plate. (**b–d**) GFP expression in spheroids 24 h after introduction of GFP pDNA and mRNA. (**b,c**) Confocal microscopy images. (**b**) GFP pDNA. (**c**) GFP mRNA. Green: GFP. Scale bars: 100 μm. (**d**) Total GFP expression levels evaluated by fluorescence measurement of cell lysates. *n* = 6. Data are presented as mean ± standard error of the mean. Statistical analysis was performed by unpaired 2-tailed Student’s *t*-test.

Human MSCs, a frequently used and highly potent source of cells in transplantation therapy, were selected for spheroid preparation [31]. MSC spheroids with a uniform size of 100 μm were successfully prepared by seeding a large number of MSCs onto a micropatterned plate (Figure 1a), comprising an array of 100-μm-sized cell adhesive areas [25]. Firstly, MSC spheroids were transfected with mRNA or pDNA encoding Gaussia luciferase (GLuc) using PAsp(DET) polycation, for evaluating time-dependent expression of GLuc protein. The level of GLuc protein expression from mRNA became maximum at 24 h post transfection, while that from pDNA reached a plateau at 24 h post transfection (Supplementary Figure S1). Thus, it is reasonable to use the observation time point of 24 h post transfection in the following experiments. Subsequently, spheroids were transfected with pDNA or mRNA encoding green fluorescence protein (GFP). After 24 h of transfection, mRNA-induced GFP expression was noted in a larger percentage of cells compared to pDNA by confocal microscopic observation of spheroids (Figure 1b,c). mRNA also yielded enhanced total GFP expression level in fluorescence measurements of cell lysate, compared to pDNA (Figure 1d).

**Figure 2:**
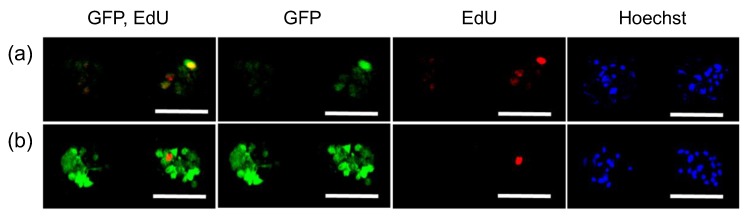
Relation between GFP expression and cell division in MSC spheroids. GFP pDNA (**a**) and GFP mRNA (**b**) were added to MSC spheroids, followed by 24 h incubation with EdU. Then, cells were observed with confocal laser scanning microscopy. Green: GFP (immunostaining), Red: EdU, Blue: cell nuclei (Hoechst 33342). Scale bars: 100 μm.

To explain the difference in the GFP expression profile observed between mRNA and pDNA transfection, we focused on the relationship between GFP expression and cell division. Dividing cells were labeled with 5-ethynyl-2’-deoxyuridine (EdU), a nucleic acid analogue, incorporated into the nuclear DNA of dividing cells during S phase [32]. Notably, only 16% of cells became EdU-positive in spheroids after 24 h of incubation, whereas the percentage of EdU-positive cells in the monolayer culture was 69%. In confocal microscopic observation after pDNA transfection with *GFP*, GFP protein expression was observed mostly in EdU-positive cells (Figure 2a). In sharp contrast, both EdU-positive and negative cells expressed GFP after mRNA transfection (Figure 2b). This observation was also supported by quantitative analyses (Table 1). As a result, the total percentage of GFP-positive cells after mRNA transfection was higher than that following pDNA transfection, presumably because of mRNA capability to induce protein expression in non-dividing cells.

**Figure 3:**
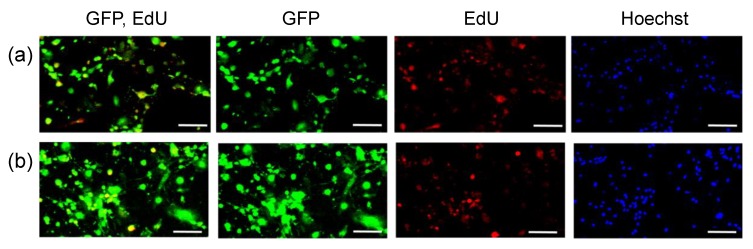
Relationship between GFP expression and cell division in MSCs in monolayer culture. GFP pDNA (**a**) and GFP mRNA (**b**) were added to MSC spheroids, followed by 24 h incubation with EdU. Then, cells were observed with fluorescence microscopy. Green: GFP, Red: EdU, Blue: cell nuclei (Hoechst 33342). Scale bars: 100 μm.

Conversely, pDNA transfection resulted in GFP expression in a large percentage of MSCs in the monolayer culture (Figure 3, Table 2), which divided more frequently than MSCs in spheroids. However, even in the monolayer culture, the percentage of GFP-positive cells in EdU-negative cells following pDNA transfection was low, as was observed in the transfection of spheroids. These results indicate that pDNA transfection is inefficient in non-dividing cells. In contrast, mRNA transfection of MSCs in the monolayer culture allowed for GFP expression in both EdU-positive and negative cells.

Finally, mRNA transfection was applied to in vitro osteogenic differentiation of MSC spheroids. pDNA and mRNA encoding runt-related transcription factor 2 (Runx2), a strong activator of osteogenic differentiation [33,34,35], was introduced to MSC spheroids four times every 3 days. Osteogenic differentiation of MSCs was evaluated by measuring the enzymatic activity of alkaline phosphatase (ALP) and the level of osteocalcin transcript, well-established markers for osteogenesis [33,34]. Runx2 mRNA transfection resulted in significantly enhanced levels of ALP compared to Runx2 pDNA transfection and untreated control spheroids, 14 days after the first transfection. Furthermore, osteocalcin transcript level was shown to be significantly increased 21 days after Runx2 mRNA introduction compared to that in untreated control spheroids, while Runx2 pDNA induced only a modest increase in *osteocalcin* expression compared to the untreated control spheroid, which was not statistically significant. These results suggest that mRNA may have enhanced capability for in vitro cell differentiation compared to pDNA.

## 4. Discussion

In this study, using MSC spheroids, we revealed that compared with pDNA transfection, mRNA transfection induced protein expression in a larger percentage of 3D cultured cells (Figure 1). High efficiency of mRNA transfection in MSC spheroids may be attributed to mRNA transfectability in non-dividing cells, a major component of spheroids. Indeed, when dividing cells were labeled with EdU, a nucleic acid analogue, GFP mRNA provided GFP protein expression both in EdU-positive and negative cells, while most of the EdU-negative cells failed to express GFP protein after *GFP* pDNA transfection (Figure 2). 

Interestingly, a small but certain number of the EdU-negative cells became GFP-positive after pDNA transfection. This result could be explained by pDNA transfection in non-dividing cells, which occurs at a low efficiency [22,36]. The result might also be attributed to the labeling method of dividing cells; nuclear transport of pDNA is expected to occur in M phase via disappearance of nuclear membrane [21], while EdU staining labeled the cells in S phase. Thus, GFP expression in EdU-negative cells after pDNA introduction may be derived from cells that passed through M phase but not through S phase during the experimental period. Despite this issue, our results revealed that the percentage of GFP positive cells among EdU-negative cells was largely different between mRNA and pDNA introduction (Figure 2 and Figure 3, Table 1 and Table 2).

**Figure 4:**
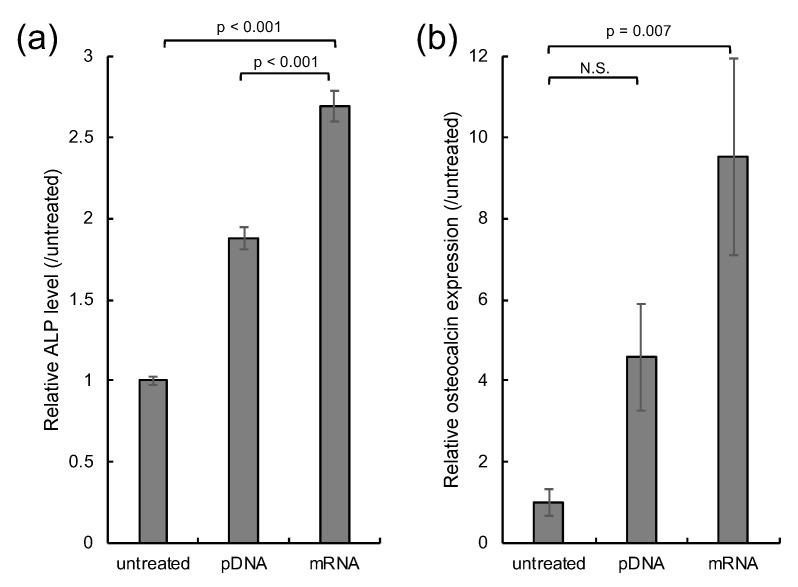
In vitro osteogenic differentiation. MSC spheroids were transfected with Runx2 mRNA and pDNA four times every 3 days. Spheroids without transfection were used as a control (untreated). (**a**) Enzymatic activity of ALP 14 days after the first transfection. *n* = 6. (**b**) Osteocalcin transcript level 21 days after the first transfection. *n* = 5. Data are presented as mean ± standard error of the mean. Statistical analysis was performed by analysis of variance (ANOVA), followed by Tukey’s test.

This study also demonstrated potential utility of mRNA transfection for ex vivo cell differentiation in cell transplantation therapy. Introduction of Runx2 mRNA allowed osteogenic differentiation of MSCs in spheroids more efficiently compared to that of Runx2 pDNA (Figure 4). Notably, mRNA transfection led to protein expression in a larger percentage of cells (Figure 1b,c), and also in a larger total quantity (Figure 1d) in spheroids, compared to pDNA transfection, and therefore, both of these mechanisms may explain the better outcome of mRNA in osteogenic differentiation. Discrimination between these two possible mechanisms would be interesting for future study. It is also important to note that the mRNA transfection reagent is another determinant of successful cell differentiation. Particularly in applications to regenerative therapy, toxicological properties of the reagent may impair functionality of the cells in exerting therapeutic effects [37,38,39]. In this regard, the biodegradability of PAsp(DET) allowed us to prevent cumulative cytotoxicity [29], which might contribute to effective osteogenic differentiation in the present study. Emerging advanced technologies of mRNA transfection will further improve the mRNA potential for ex vivo cell differentiation [40,41,42,43,44,45]. Conclusively, mRNA exhibits high potential in gene transfection to 3D cell culture, in which the cell division rate is lower than that in monolayer cultures. For future application of our technology to bone regeneration therapy, further detailed in vitro evaluations, including Alizarin Red and Von Kossa staining, and in vivo transplantation study are needed. In addition, ex vivo generation of other cell types will be performed in the future to demonstrate the versatility of our approach in regenerative therapy.

## Figures and Tables

**Table 1:** Percentage of GFP-positive cells in MSC spheroids.

	EdU (+) ^1^	EdU (−) ^2^	Total ^3^
GFP pDNA (%) ^4^	75	20	34
GFP mRNA (%) ^4^	100	73	77

^1−3^ Percentage of GFP-positive cells in EdU-positive cells ^1^, EdU-negative cells ^2^, and all cells ^3^ in MSC spheroids. ^4^ More than 150 cells in 4 wells were evaluated for each group.

**Table 2:** Percentage of GFP-positive cells in MSCs in monolayer culture.

	EdU (+) ^1^	EdU (−) ^2^	Total ^3^
*GFP* pDNA (%) ^4^	90	32	75
*GFP* mRNA (%) ^4^	97	85	93

^1–3^ Percentage of GFP-positive cells in EdU-positive cells ^1^, EdU-negative cells ^2^, and all cells ^3^ in MSCs in monolayer culture. ^4^ More than 300 cells in 4 wells were evaluated for each group.

## Supplementary Materials

The following are available online at https://www.mdpi.com/2072-666X/11/4/426/s1, Figure S1: Time dependent profile of transfection efficiency.

## References

[1] Trounson A., McDonald C. (2015). Stem Cell Therapies in Clinical Trials: Progress and Challenges. Cell Stem Cell.

[2] Dimmeler S., Ding S., Rando T.A., Trounson A. (2014). Translational strategies and challenges in regenerative medicine. Nat. Med..

[3] Clarke G., Harley P., Hubber E.L., Manea T., Manuelli L., Read E., Watt F.M. (2018). Bench to bedside: Current advances in regenerative medicine. Curr. Opin. Cell Biol..

[4] Fox I.J., Daley G.Q., Goldman S.A., Huard J., Kamp T.J., Trucco M. (2014). Stem cell therapy. Use of differentiated pluripotent stem cells as replacement therapy for treating disease. Science.

[5] Meyerrose T., Olson S., Pontow S., Kalomoiris S., Jung Y., Annett G., Bauer G., Nolta J.A. (2010). Mesenchymal stem cells for the sustained in vivo delivery of bioactive factors. Adv. Drug Deliv. Rev..

[6] Masuda S., Shimizu T. (2016). Three-dimensional cardiac tissue fabrication based on cell sheet technology. Adv. Drug Deliv. Rev..

[7] Vijayavenkataraman S., Yan W.C., Lu W.F., Wang C.H., Fuh J.Y.H. (2018). 3D bioprinting of tissues and organs for regenerative medicine. Adv. Drug Deliv. Rev..

[8] June C.H., O’Connor R.S., Kawalekar O.U., Ghassemi S., Milone M.C. (2018). CAR T cell immunotherapy for human cancer. Science.

[9] De Ravin S.S., Li L., Wu X., Choi U., Allen C., Koontz S., Lee J., Theobald-Whiting N., Chu J., Garofalo M. (2017). CRISPR-Cas9 gene repair of hematopoietic stem cells from patients with X-linked chronic granulomatous disease. Sci. Transl. Med..

[10] Sheyn D., Mizrahi O., Benjamin S., Gazit Z., Pelled G., Gazit D. (2010). Genetically modified cells in regenerative medicine and tissue engineering. Adv. Drug Deliv. Rev..

[11] Zhang K., Fang H., Qin Y., Zhang L., Yin J. (2018). Functionalized Scaffold for in Situ Efficient Gene Transfection of Mesenchymal Stem Cells Spheroids toward Chondrogenesis. ACS Appl. Mater. Interfaces.

[12] Song S.Y., Hong J., Go S., Lim S., Sohn H.S., Kang M., Jung G.J., Yoon J.K., Kang M.L., Im G.I. (2020). Interleukin-4 Gene Transfection and Spheroid Formation Potentiate Therapeutic Efficacy of Mesenchymal Stem Cells for Osteoarthritis. Adv. Healthc. Mater..

[13] Uchida S., Hayakawa K., Ogata T., Tanaka S., Kataoka K., Itaka K. (2016). Treatment of spinal cord injury by an advanced cell transplantation technology using brain-derived neurotrophic factor-transfected mesenchymal stem cell spheroids. Biomaterials.

[14] Yanagihara K., Uchida S., Ohba S., Kataoka K., Itaka K. (2018). Treatment of Bone Defects by Transplantation of Genetically Modified Mesenchymal Stem Cell Spheroids. Mol. Ther.-Methods Clin. Dev..

[15] Kaiser J. (2020). How safe is a popular gene therapy vector?. Science.

[16] Qin L., Ding Y., Pahud D.R., Robson N.D., Shaked A., Bromberg J.S. (1997). Adenovirus-mediated gene transfer of viral interleukin-10 inhibits the immune response to both alloantigen and adenoviral antigen. Hum. Gene Ther..

[17] Levine B.L., Miskin J., Wonnacott K., Keir C. (2017). Global Manufacturing of CAR T Cell Therapy. Mol. Ther. Methods Clin. Dev..

[18] Ran F.A., Cong L., Yan W.X., Scott D.A., Gootenberg J.S., Kriz A.J., Zetsche B., Shalem O., Wu X., Makarova K.S. (2015). In vivo genome editing using Staphylococcus aureus Cas9. Nature.

[19] Freyer J.P. (1988). Role of necrosis in regulating the growth saturation of multicellular spheroids. Cancer Res..

[20] Onozato Y., Kaida A., Harada H., Miura M. (2017). Radiosensitivity of quiescent and proliferating cells grown as multicellular tumor spheroids. Cancer Sci..

[21] Capecchi M.R. (1980). High efficiency transformation by direct microinjection of DNA into cultured mammalian cells. Cell.

[22] Grosse S., Thevenot G., Monsigny M., Fajac I. (2006). Which mechanism for nuclear import of plasmid DNA complexed with polyethylenimine derivatives?. J. Gene Med..

[23] Zou S., Scarfo K., Nantz M.H., Hecker J.G. (2010). Lipid-mediated delivery of RNA is more efficient than delivery of DNA in non-dividing cells. Int. J. Pharm..

[24] Sahin U., Kariko K., Tureci O. (2014). mRNA-based therapeutics—Developing a new class of drugs. Nat. Rev. Drug Discov..

[25] Otsuka H., Hirano A., Nagasaki Y., Okano T., Horiike Y., Kataoka K. (2004). Two-dimensional multiarray formation of hepatocyte spheroids on a microfabricated PEG-brush surface. Chembiochem.

[26] Uchida S., Itaka K., Nomoto T., Endo T., Matsumoto Y., Ishii T., Kataoka K. (2014). An injectable spheroid system with genetic modification for cell transplantation therapy. Biomaterials.

[27] Kanayama N., Fukushima S., Nishiyama N., Itaka K., Jang W.D., Miyata K., Yamasaki Y., Chung U.I., Kataoka K. (2006). A PEG-based biocompatible block catiomer with high buffering capacity for the construction of polyplex micelles showing efficient gene transfer toward primary cells. ChemMedChem.

[28] Miyata K., Oba M., Nakanishi M., Fukushima S., Yamasaki Y., Koyama H., Nishiyama N., Kataoka K. (2008). Polyplexes from poly(aspartamide) bearing 1,2-diaminoethane side chains induce pH-selective, endosomal membrane destabilization with amplified transfection and negligible cytotoxicity. J. Am. Chem. Soc..

[29] Itaka K., Ishii T., Hasegawa Y., Kataoka K. (2010). Biodegradable polyamino acid-based polycations as safe and effective gene carrier minimizing cumulative toxicity. Biomaterials.

[30] Matsui A., Uchida S., Ishii T., Itaka K., Kataoka K. (2015). Messenger RNA-based therapeutics for the treatment of apoptosis-associated diseases. Sci. Rep..

[31] Galipeau J., Sensebe L. (2018). Mesenchymal Stromal Cells: Clinical Challenges and Therapeutic Opportunities. Cell Stem Cell.

[32] Salic A., Mitchison T.J. (2008). A chemical method for fast and sensitive detection of DNA synthesis in vivo. Proc. Natl. Acad. Sci. USA.

[33] Banerjee C., McCabe L.R., Choi J.Y., Hiebert S.W., Stein J.L., Stein G.S., Lian J.B. (1997). Runt homology domain proteins in osteoblast differentiation: AML3/CBFA1 is a major component of a bone-specific complex. J. Cell. Biochem..

[34] Zhao Z., Zhao M., Xiao G., Franceschi R.T. (2005). Gene transfer of the Runx2 transcription factor enhances osteogenic activity of bone marrow stromal cells in vitro and in vivo. Mol. Ther..

[35] Liu T.M., Lee E.H. (2013). Transcriptional regulatory cascades in Runx2-dependent bone development. Tissue Eng Part B Rev.

[36] Akita H., Kurihara D., Schmeer M., Schleef M., Harashima H. (2015). Effect of the Compaction and the Size of DNA on the Nuclear Transfer Efficiency after Microinjection in Synchronized Cells. Pharmaceutics.

[37] Uchida S., Kataoka K. (2019). Design concepts of polyplex micelles for in vivo therapeutic delivery of plasmid DNA and messenger RNA. J. Biomed. Mater. Res. A.

[38] Endo T., Itaka K., Shioyama M., Uchida S., Kataoka K. (2012). Gene transfection to spheroid culture system on micropatterned culture plate by polyplex nanomicelle: A novel platform of genetically-modified cell transplantation. Drug Deliv Transl Res.

[39] Masago K., Itaka K., Nishiyama N., Chung U.I., Kataoka K. (2007). Gene delivery with biocompatible cationic polymer: Pharmacogenomic analysis on cell bioactivity. Biomaterials.

[40] McKinlay C.J., Vargas J.R., Blake T.R., Hardy J.W., Kanada M., Contag C.H., Wender P.A., Waymouth R.M. (2017). Charge-altering releasable transporters (CARTs) for the delivery and release of mRNA in living animals. Proc. Natl. Acad. Sci. USA.

[41] Hajj K.A., Whitehead K.A. (2017). Tools for translation: Non-viral materials for therapeutic mRNA delivery. Nat. Rev. Mater..

[42] Zhong Z., Mc Cafferty S., Combes F., Huysmans H., De Temmerman J., Gitsels A., Vanrompay D., Portela Catani J., Sanders N.N. (2018). mRNA therapeutics deliver a hopeful message. Nano Today.

[43] Li J., Wang W., He Y., Li Y., Yan E.Z., Zhang K., Irvine D.J., Hammond P.T. (2017). Structurally Programmed Assembly of Translation Initiation Nanoplex for Superior mRNA Delivery. ACS Nano.

[44] Yoshinaga N., Cho E., Koji K., Mochida Y., Naito M., Osada K., Kataoka K., Cabral H., Uchida S. (2019). Bundling mRNA Strands to Prepare Nano-Assemblies with Enhanced Stability Towards RNase for in Vivo Delivery. Angew. Chem. Int. Ed..

[45] Yoshinaga N., Uchida S., Naito M., Osada K., Cabral H., Kataoka K. (2019). Induced packaging of mRNA into polyplex micelles by regulated hybridization with a small number of cholesteryl RNA oligonucleotides directed enhanced in vivo transfection. Biomaterials.

