# Supplementary material for: mRNA as a Tool for Gene Transfection in 3D Cell Culture for Future Regenerative Therapy
Source: Micromachines (Basel). 2020 Apr 18;11(4):426. doi: 10.3390/mi11040426 (PMC7231348; doi:10.3390/mi11040426)
Supplement: Supplementary file 1 [file micromachines-11-00426-s001.pdf]

Supplementary Materials: mRNA as a Tool for Gene Transfection in 3D Cell Culture for Future Regenerative Therapy

Satoshi Uchida, Kayoko Yanagihara, Akitsugu Matsui, Kazunori Kataoka and Keiji Itaka

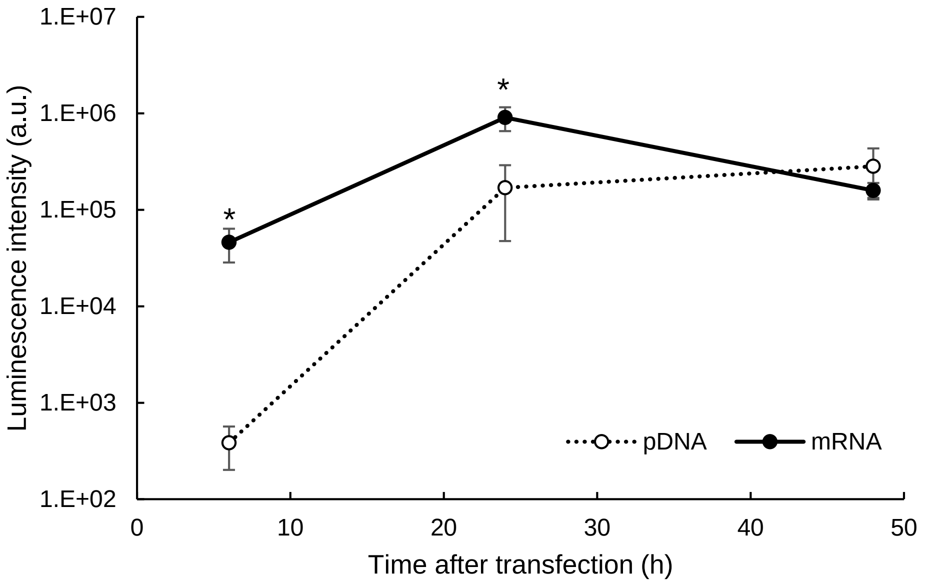

Figure S1. Time dependent profile of transfection efficiency. MSC spheroids were transfected with mRNA and pDNA encoding *Gaussia luciferase* (*GLuc*). A coding sequence of *GLuc* in a pCMV-GLuc control vector (New England BioLabs, Ipswich, MA, USA) was inserted into pSP73 vector (Promega, Madison, WI, USA) possessing a 120 bp poly A/T sequence for preparation of mRNA by *in vitro* transcription, and into pCAG-GS vectors (RIKEN, Tokyo, Japan) for *GLuc* pDNA construction. Note that both mRNA and pDNA have an identical coding sequence of *GLuc* (Genbank: MO429382.1). Subsequently, MSC spheroids cultured in 96-well micropatterned plates were transfected with *GLuc* mRNA or pDNA complexed with PAsp(DET) at N/P ratio of 10, as described in Section 2.3. At indicated time point, culture medium was collected for luminescence measurement using Renilla Luciferase Assay System (Promega) and a GloMax-Multi+ Detection System (Promega, Madison, WI, USA), followed by replacement of the medium with the fresh one. $n = 4$. Data are presented as mean ± standard error of the mean. Statistical analysis was performed by unpaired 2-tailed Student's *t*-test. * $p < 0.05$ vs. pDNA.
